# Characterization and Caco-2 Cell Transport Assay of Chito-Oligosaccharides Nano-Liposomes Based on Layer-by-Layer Coated

**DOI:** 10.3390/molecules26144144

**Published:** 2021-07-07

**Authors:** Tingting Cui, Airong Jia, Mengke Yao, Miansong Zhang, Chanchan Sun, Yaping Shi, Xue Liu, Jimin Sun, Changheng Liu

**Affiliations:** 1 Biology Institute, Qilu University of Technology (Shandong Academy of Sciences), Jinan 250103, China; tingtingcui@sdas.org (T.C.); YMK970530@126.com (M.Y.); zhangms@sdas.org (M.Z.); shiyp@sdas.org (Y.S.); liuxue@sdas.org (X.L.); 15866708263@126.com (J.S.); liuchh@sdas.org (C.L.); 2 China-Australia Joint Laboratory for Native Bioresource Industry Innovation, Qilu University of Technology (Shandong Academy of Sciences), Jinan 250103, China; 3 College of Life Sciences, Yantai University, Yantai 264005, China; sunchan88@126.com; 4 Key Laboratory of Food Nutrition and Safety (Tianjin University of Science &Technology), Ministry of Education, Tianjin 300457, China

**Keywords:** chito-oligosaccharides (COSs), chitosan (CH), sodium alginate (SA), nano-liposomes, Caco-2 cell transport

## Abstract

Chito-oligosaccharides (COSs) were encapsulated by the film-ultrasonic method into three nano-liposomes, which were uncoated liposomes (COSs-Lip), chitosan-coated liposomes (CH-COSs-Lip), and sodium alginate (SA)/chitosan (CH)-coated liposomes (SA/CH-COSs-Lip). The physicochemical and structural properties, as well as the stability and digestive characteristics, of all three nano-liposomes were assessed in the current study. Thereafter, the characteristics of intestinal absorption and transport of nano-liposomes were investigated by the Caco-2 cell monolayer. All nano-liposomes showed a smaller-sized distribution with a higher encapsulation efficiency. The ζ-potential, Z-average diameter (Dz), and polydispersity index (PDI) demonstrated that the stability of the SA/CH-COSs-Lip had much better stability than COSs-Lip and CH-COSs-Lip. In addition, the transport of the nano-liposomes via the Caco-2 cell monolayer indicated a higher transmembrane transport capacity. In summary, the chitosan and sodium alginate could serve as potential delivery systems for COSs to fortify functional foods and medicines.

## 1. Introduction

Chito-oligosaccharide (COS) is a kind of water-soluble cationic amino polysaccharide. Being made of glucosamine, COSs are linked by β-1,4-glycosidic linkages and obtained from the degradation of shrimp and crab shells. Its degree of polymerization (DP) is less than 20%, and molecular weight (*M*_w_) is generally less than 5 kDa [1,2,3]. In addition, COSs has several physicochemical properties, including low viscosity, high water-solubility, and non-toxicity, thus providing a great potential in medical, pharmacological, and industrial applications [4,5,6,7].

Previously, many studies have proved that COSs exhibit an enormously wide range of biological activities, including antitumor, anti-bacteria, anti-fungus, antioxidant, anti-inflammation, immunomodulation, and so forth [8,9,10]. They also have a remarkable potential to be applied in pharmacological and biomedical fields. Compared with chitosan and chitin, COSs are equipped with reactive functional groups, such as hydroxyl groups at C3 and C6, while the amino and *N*-glucosamine units are linked by glycosides [11,12]. These properties are crucial for COSs’ natural biological effects and applicable utilization. 

A liposome is an artificial vesicle composed of one or more concentric phospholipid bilayers and used specially to deliver microscopic substances, such as drugs or DNA, to body cells [13,14]. Liposomes are usually used as carriers to protect some functional components, which could improve the bioavailability, stability, and shelf life of components. Preparation of COSs’ liposomes exhibited favorable biocompatibility, targeting ability, and release properties. Moreover, COSs’ liposomes can improve the efficiency of COSs passing through the cell membrane and increase their bioavailability.

During storage, however, liposomes’ structure is easily damaged by light, acid, and alkali, leading to problems such as flocculation, particles becoming larger, and drug-release irregularity. In addition, liposomes are often used in oral preparations, but it can be easily affected by acids and enzymes, while being absorbed and digested in the body [15,16]. This causes hydrolysis to occur in the liposomes’ phospholipid wall and damage to the phospholipids bilayers, leading to the leak of embedded materials. All of those issues have limited the application of liposomes.

According to previous studies, the modification on COSs’ liposomes surface can improve its stability, and there are a lot of reports on the modification of liposomes with single polymers such as chitosan, polyethylene glycol, and protein [17,18]. However, during the modification process, the binding force and structure between the COSs’ liposomes and the modification layer is week, and the storage stability of the monolayer modified liposomes is not ideal. Therefore, two or more coated-layers (layer-by-layer coated) are needed to improve the stability of COSs’ liposomes [19,20]. In addition, layer-by-layer coated COSs’ liposomes could reduce the digestibility of liposomes, prolong circulation time, and have a better medicine effect in the body.

Chitosan and sodium alginate, as natural polycationic and polyanionic polysaccharides, which have good biocompatibility, biodegradability and adhesion, and widely used in embedding active ingredients [21,22]. Chitosan in acidic solution is cationic, so it can integrate with negatively charged liposomes through electrostatic interaction, in order to form a protective film on the surface of liposomes. Previous studies from Hanafy et al. [23] designed the promising mucoadhesive delivery system for encapsulating curcumin LbL nano-template with chitosan, which could fight against the invasiveness of breast cancer. It is generally believed that chitosan-coated nano-template is a potential carrier for embedding other functional components with biological activities, which can be used in processing and producing functional foods. Haidar et al. [24] have modified bovine serum protein liposome with sodium alginate and chitosan, in order to test its storage stability. The research results show that the stability of liposomes can be greatly enhanced when coated with sodium alginate–chitosan bilayers.

Caco-2 cells were derived from human colon cancer cells and they can spontaneously differentiate into intestinal-cell-like cells under the conventional cell culture conditions. Moreover, these cells’ morphology is very similar to human intestinal epithelial cells, and they have same cellular polarity. Therefore, Caco-2 cells can be used as a model to study the intestinal permeability and transport mechanism of liposomes [25].

In this study, the COSs’ liposome was coated by chitosan and sodium alginate. The morphology and structure of nano-liposomes were characterized by TEM, and the ζ-potential, Z-average diameter (Dz), polydispersity index (PDI), and encapsulation efficiency were also tested. Finally, the transport rate of the nano-liposome onto the Caco-2 cell monolayer assay was evaluated to determine the transport mechanism of the nano-liposomes. This study provides comprehensive technical support to prove that the stability, bioavailability, and transshipment of COSs’ nano-liposomes are improved with chitosan and sodium alginate. Therefore, the study results could help to expand the field of COSs’ study and applications.

## 2. Materials and Methods

### 2.1. Materials

Soybean Lecithin, cholesterol, and Tween 80 were purchased from Solaibao Biological Technology Co., Ltd. (Beijing, China). Chito-oligosaccharide (*M*_w_ < 3000 Da), chitosan (CH, *M*_w_ = 2.31 × 10^5^ Da), and sodium alginate (SA, *M*_w_ = 1.18 × 10^5^ Da) were provided by the Yuanye Biotechnology Co., Ltd. (Shanghai, China). Caco-2 cell lines were obtained from Dingguo Changsheng Biotechnology Co., Ltd. (Beijing, China). Other reagents and chemicals were of analytical purity, and deionized distilled water was used in all experiments.

### 2.2. Preparation of Liposomes

**Figure 1:**
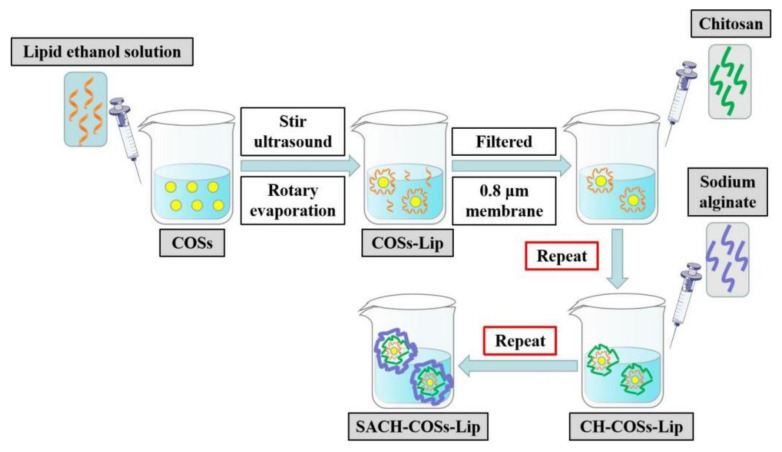
Preparation of nano-liposomes.

The chito-oligosaccharides nano-liposomes (COSs-Lip) were prepared by the film-ultrasonic method (Figure 1). Briefly, soybean lecithin, cholesterol, and Tween 80 (4:1:1.6, wt%) were dissolved in 20 mL of absolute ethyl alcohol to form uniform transparent film. Then 80 mg of COSs was dissolved in 20 mL phosphate buffer (0.05 mol/L, pH 6.0). Afterwards, the lipids and COSs were thoroughly mixed after 30 min magnetic stirring at a temperature of 25 °C, the solvent was removed by rotary evaporation at 50 °C, and the layer of viscous gel was dried in a vacuum oven overnight. The thin film was hydrated with phosphate buffer (0.05 mol/L, pH 6.0) by stirring for 1 h and then ultrasonic treatment for 10 min. Then the COSs-Lip solution was stored at 4 °C before use.

**Figure 2:**
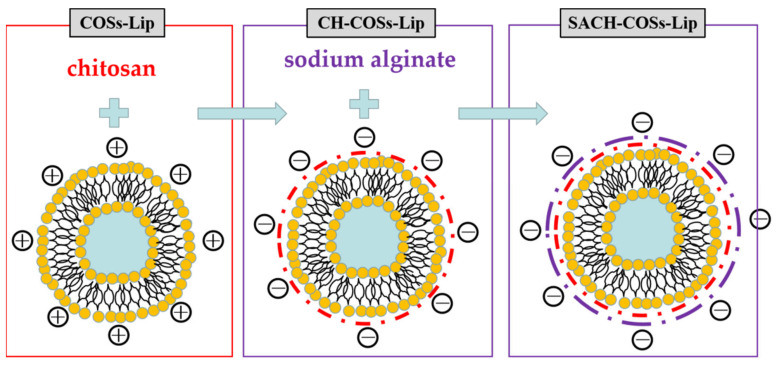
Chemical interaction during the preparation of chito-oligosaccharides nano-liposomes by layer-by-layer coated with chitosan and sodium alginate.

The chitosan-coated COSs-Lip (CH-COSs-Lip) was prepared by electrostatic interaction between negatively charged COSs-Lip and positively charged chitosan (Figure 2). The COSs-Lip suspension (1 mg/mL) was mixed with an equal volume of chitosan solution (0.5% in citrate buffer solution, pH 4.0) under 30 min stirring and then the suspension stands for 2 h. Then the chitosan can fully coat the surface of COSs-Lip, making the chitosan-coated liposomes (CH-COSs-Lip).

For the preparation of bilayer-coasted COSs-Lip (SA/CH-COSs-Lip), sodium alginate (0.1% in phosphate buffer, pH 6.0) was mixed with an equal volume of CH-Lip (1 mg/mL) under 30 min stirring, and the suspension stands for 2 h. Then the sodium alginate can fully coat the surface of CH-Lip, making the sodium alginate (SA)/chitosan (CH)-coated liposomes (SA/CH-COSs-Lip).

### 2.3. Characterization of the Liposomes

#### 2.3.1. Determination of Encapsulation Efficiency

The encapsulation efficiency of liposomes was determined by ultraviolet spectroscopy in this experiment. A 100 μL liposomes suspension (1 mg/mL) was diluted with distilled water, and mixed in a 10 mL volumetric flask. The solution was centrifuged at 10,000 rpm for 30 min, and the supernatant was collected. The content of free oligosaccharide (*E*1) in supernatant was determined by phenol-sulfuric acid method, with some modification, according to Masuko [26], and blank liposomes served as the control. 

We took a 1 mL liposomes suspension (1 mg/mL) and blank liposomes suspension in 10 mL volumetric flask and added absolute ethyl alcohol for demulsification. The content of total oligosaccharide content (*E2*) was determined by phenol-sulfuric acid method, and blank liposomes served as control. The encapsulation efficiency was calculated by using Equation (1):

(1) Encapsulation efficiency=(E2−E1)/E2×100%

#### 2.3.2. Transmission Electron Microscopy (TEM) Analysis

Transmission electron microscopy (TEM) was used to observe liposomes’ morphology. The samples were prepared by adopting a negative staining method with phosphotungstic acid aqueous solution (2.0%). Briefly, one drop of nanogel dispersion was placed on a carbon formvar-coated copper grid (200 mesh) for 5 min, and the excess solution was wiped away by filter paper to form a thin liquid film on the copper grid. Next, one drop of phosphotungstic acid aqueous solution was placed on the copper grid. The excess liquid was also wiped away by filter paper, and the samples were dried in air. Then it was visualized by an HT-7800 TEM (Hitachi, Ltd., Tokyo, Japan) transmission electron microscope at an accelerating voltage of 200 kV.

#### 2.3.3. ζ-Potential, Z-Average Diameter (Dz), and Polydispersity Index (PDI) Analysis

The particle size distribution of the liposomes′ suspension, including Dz and PDI, were calculated by using a Zetasizer NanoZS90 particle size analyzer (Malvern Instruments Ltd., Malvern, United Kingdom) equipped with a He/Ne laser (λ = 633 nm) and a scattering angle of 90 °. Aliquots of 1 mL liposomes suspension were diluted in 10 mL buffer solution to avoid the multiple scattering phenomena caused by interparticle interactions. Immediately, the diluted sample (10%, *v/v*) was transferred into a polystyrene cuvette for size determination at 25 ± 0.1 °C. Thereafter, the Dz and PDI were recorded. Three parallel experiments were carried out with its sample.

The ζ-potential measurements of the liposomes′ suspension were also measured by checking the laser Doppler electrophoretic mobilities using a Zetasizer Nano ZS 90 (Malvern Instruments Ltd., Malvern, United Kingdom). The samples were appropriately diluted 10-fold with the same buffer before determination in order to avoid the multiple scattering phenomena. The mobility, u, was converted into ζ-potential by using the Smoluchowski relation, ζ = uη/ε, where η and ε were the viscosity and permittivity of the solution, respectively. Three parallel experiments were carried out with each sample, and each measurement was obtained from the mean of at least 10 readings of the sample.

#### 2.3.4. Fourier Transform Infrared (FTIR) Spectra Analysis

The study on chemical bond of liposomes was operated with Fourier transform infrared (FTIR) spectroscopy. FTIR spectra were recorded over the wavelength range of 4000 to 400 cm^−1^, by using a Nicolet IS50 FTIR spectrometer (Thermo Nicolet Corp, Madison, WI, USA) equipped with a single reflection diamond attenuated total reflectance (ATR) crystal and a mercury–cadmium–telluride (MCT) detector. A total of 1.0 mg of liposomes (freeze-dried samples) was mixed with 150 mg KBr powder and compressed into discs at a force of 5 KN for 30 s. FTIR spectra were recorded with 64 scans and a 4 cm^−1^ resolution against the background. The differences between chemical bond of liposomes samples were analyzed by the FTIR spectra.

#### 2.3.5. X-ray Diffraction (XRD) Analysis

The crystal state of liposomes was analyzed by an X-ray diffractometer (D8-Discover, Bruker, Corp, Germany) according to Zhang et al. [27]. Diffraction patterns of liposomes were recorded over a range of 2θ angles, from 2° to 90°, at a speed of 0.002 °/s. Samples were irradiated by Cu-Ka radiation, at a 30 mA current and a voltage of 40 kV.

### 2.4. In Vitro Digestive Stability

Samples of liposomes in vitro release was investigated by employing both simulated gastric fluid (SGF) and simulated intestinal fluid (SIF) model, according to a previous report by Liu et al. [28]. SGF was prepared by dissolving 2.0 g NaCl into 900 mL distilled water, and the pH of solution was adjusted to 1.2 with 36% HCl. Then 3.2 g of pepsin was added, and the solution was finally made up to 1000 mL with distilled water. SIF was prepared by dissolving 6.8 g K_2_HPO_4_ into 800 mL distilled water, followed by adding 77 mL NaOH (0.2 M); the pH value of the solution was adjusted to 6.8 with NaOH (1 M) or HCl (1 M), and 10 g trypsin was added. The volume of the solution was made up to 1000 mL with distilled water and stored at 4 °C until further analyses.

Next, the liposomes were added to digestive juice (SGF/SIF) at a 1:3 (*w/v*) ratio and dissolved thoroughly. The digestive reaction was simulated by incubation in a shaker at 37 °C and 90 r/min. Samples were taken and analyzed at regular intervals of digestion time of 0, 25, 50, 75, 100, 125, 150, 175, 200, 225, 250, 275, and 300 min, respectively. The release rate of COSs in the liposomes samples was determined by phenol-sulfuric acid method according to the Section 2.3.1, and it was calculated according to Equation (2); in addition, three parallel experiments were carried out with each sample.

(2) Release rate (%)=(Em−En)/(Etotal−En)×100%

where *Em* is the content of free COSs measured at m min (mg), *En* is the content of free COSs measured at reaction start time (mg), and *Etotal* is the total amount of COSs added for the liposomes preparation (mg).

### 2.5. Absorption Characteristic of the Liposomes in Caco-2 Cells

#### 2.5.1. MTT Assay

Liposomes′ concentrations were calculated by the Caco-2 cell effect and the MTT colorimetric assay [29]. At the logarithmic growth stage, Caco-2 cells were inoculated into 96-well plates at a density of approximately 1×10^4^ cells/well, and 150 µL of the liposomes solutions was added to each well. After 24 h incubation at 37 °C with 5% CO_2_, the supernatant was discarded, and 150 µL liposomes solutions was added to each well. At the same time, a blank control group with six parallel cells in each group were established. By 24 h later, 20 µL MTT (5 mg/mL) was added to each well and reacted for approximately 4 h. Thereafter, the supernatant was discarded, and 150 µL DMSO was added to each well and shaken for 10 min. The optical density (OD) value of each well was measured by using the ELIAS method at a wavelength of 490 nm. The Caco-2 cell survival rate was calculated by following Equation (3):

(3) $$\mathrm{Cell}\;\mathrm{survival}\;\mathrm{rate}\;(\%)=\frac{OD1-OD2}{OD3-OD2}\times 100\%$$

where *OD*1, *OD*2, and *OD*3 are the OD values of the experimental group, blank group, and culture medium, respectively.

#### 2.5.2. Caco-2 Cell Transport Assay

The liposomes solutions were filtered and sterilized by a 0.45 µm membrane respectively and preheated at 37 °C for further application. When the transepithelial electrical resistance (TEER) of Caco-2 cells is >600 Ω·cm^2^ on the transwell plate and the apparent permeability coefficient of fluorescent yellow *Papp* is <1.0 × 10^−6^ cm/s, cell transport experiments can be conducted. The Caco-2 cell layer was washed three times with HBSS solution (pH = 7.4), and the cells were placed in an incubator, at 37 °C, for 15 min, after the third wash; thereafter, the HBSS solution was removed. For transporting from the apical side (AP side) to the basolateral side (BL side), 0.5 mL of the sample solution was added to the AP side as the supply pool, and 1.5 mL of the HBSS solution was added to the BL side as the receiving pool. For transporting from the BL side to the AP side, 1.5 mL sample solution was added to the BL side as the supply pool and 0.5 mL HBSS solution was added to the AP side as the receiving pool. Up to 150 µL sample was taken for each time. Meanwhile, 150 µL of HBSS solution was added correspondingly at 37 °C. The content of COSs in each sample was determined via phenol-sulfuric acid method.

### 2.6. Statistical Analysis

Statistical data analysis of three independent replicates was performed. The data were indicated as mean and standard (SD) deviation. The results were calculated, and graphs were plotted via Origin software (version 9.1). Mean values were compared by the Tukey test, and one-way analysis of variance (ANOVA) with a significance level of 0.05 (*p* < 0.05) was carried out.

## 3. Results

### 3.1. Characterization of the Liposomes

#### 3.1.1. Encapsulation Efficiency

Encapsulation efficiency is an important index to evaluate the quality and application value of nano-liposomes, which can reflect the retention rate of COSs in liposomes system during the preparation process. As seen from the Table 1, the encapsulation efficiency of COSs for COSs-Lip, CH-COSs-Lip, and SA/CH-COSs-Lip was (61.23 ± 2.09) %, (82.34 ± 4.53) %, (94.45 ± 3.08) %, respectively. This illustrated that, after being coated by CH and layer-by-layer coated by CH/SA, the encapsulation efficiency of these two COSs liposomes respectively increased by 21.11% and 33.22%. There were two possibilities for the increase of the encapsulation efficiency. First, while the liposomes were coated by CH/SA, free COSs were also embedded on samples′ surface. Second, chitosan and sodium alginate formed a dense layer to coat liposomes, which avoided the release of COSs. Moreover, Liu et al. [28] implied that the encapsulation efficiency of liposomes was greatly increased after being multilayer coated by chitosan and sodium alginate, which was similar to the result of this study.

#### 3.1.2. Dz, PDI, and ζ-Potential

The size of liposomes directly impacts on the embedding materials’ capacity, release amount, bioavailability and distribution. Controlling the size of particles and obtaining a narrow and similar particle distribution were the key to prepare nano-liposomes. Table 1 showed that the Dz of COSs, COSs-Lip, CH-COSs-Lip, and SA/CH-COSs-Lip was 187.30, 201.44, 256.47, and 331.19 nm, respectively. The Dz of liposomes increased after being prepared from COSs: the Dz of COSs-Lip increased by 55.03 nm and 74.72 nm, after being coated by CH modification and layer-by-layer coated by CH/SA, respectively. This was preliminary proved that chitosan and sodium alginate were coated on the surface of liposomes. In addition, compared with the PDI of COSs-Lip, the PDI of CH-COSs-Lip and SA/CH-COSs-Lip increased, but its value was still less than 0.5. This may be because the modification of chitosan and sodium alginate on the outer layer of the liposomes made the surface of the liposomes rough, and some uncoated chitosan and sodium alginate made the distribution of the whole system more dispersed. Almalik et al. [30] found that, after they coated liposomes with chitosan, the Dz and PDI were also increased, which was similar to the results of this current study.

**Figure 3:**
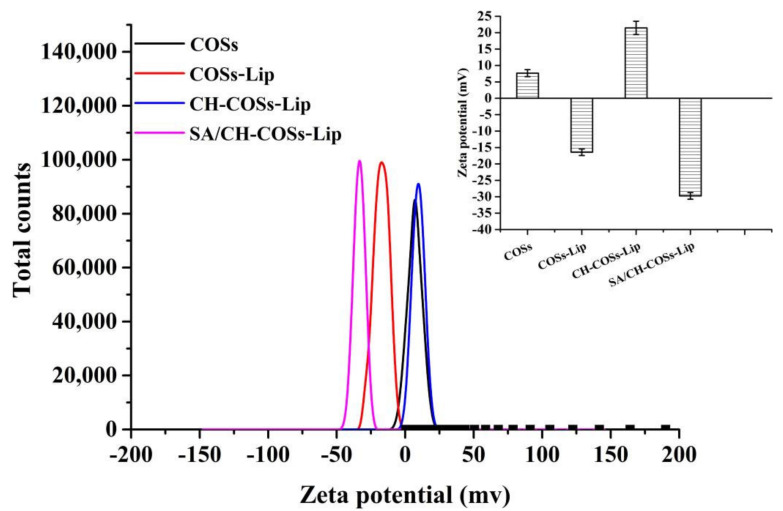
ξ-potential of COSs and COSs nano-liposomes.

ζ-potential can measure the potential charge, and the presence of modifier can change the charged condition on the surface of nano-liposomes. Analysis on the potential value of nano-liposomes before and after modification can judge whether the modifier was successfully coated on the liposomes [31]. As Figure 3 shows, the ζ-potential of COSs was 7.68 mV, and that of COSs-Lip was −16.44 mV. The figure changed to 22.04 mV after being coated with chitosan modification, and to −29.75 mV after being coated with sodium alginate. The phosphate groups make the ζ-potential of COSs-Lip to a negative value, while chitosan carries a large amount of positive charge. When chitosan was wrapped on the surface of COSs-Lip, the ζ-potential value changes to positive. Similarly, Zhou et al. [32] also found that when chitosan was used to coat Lip, the ζ-potential of Lip increased with the increase of the amount of chitosan. Besides, sodium alginate molecules with a large amount of negative charge can also be tightly wrapped on the surface of chitosan through electrostatic action, so that the ζ-potential of chitosan-coated Lip changes from positive to negative. The change of ζ-potential further confirmed that chitosan and sodium alginate were successfully coated on the surface of Lip by electrostatic adsorption.

#### 3.1.3. Microstructure by TEM Analysis

**Figure 4:**
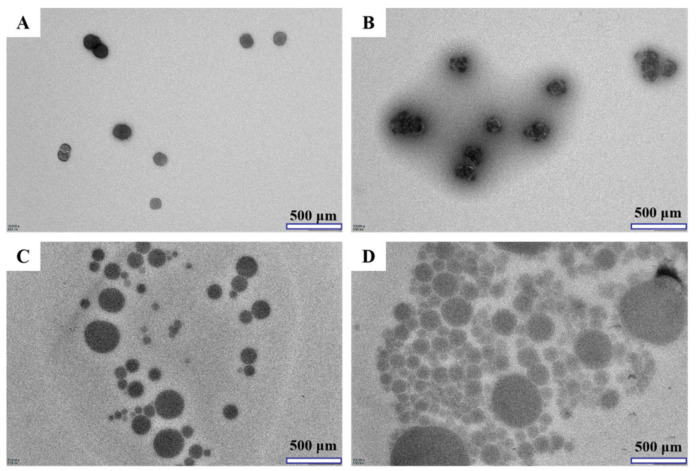
Transmission electron micrographs of COSs and COSs nano-liposomes (×10,000). (**A**) COSs. (**B**) COSs-Lip. (**C**) CH-COSs-Lip. (**D**) SA/CH-COSs-Lip.

As can be seen from Figure 4A,B, the appearance of the COSs was similar with COSs-Lip, which also presented spherical or near-spherical, and the particle size of nano-liposomes became larger. As shown in Figure 4C, COSs-Lip was coated with chitosan and obtained the CH-COSs-Lip, which was still presented a spherical particle, but it covered an obvious layer of transparent materials, and speculated the transparent materials was the chitosan. Besides, it was consistent with previous studies [33]. SA/CH-COSs-Lip (Figure 4D) was also spherical, and there is a ring of light material on the outer layer, which should be sodium alginate covering the outer layer of CH-COSs-Lip. Moreover, the SA/CH-COSs-Lip particle size was smaller and similar.

#### 3.1.4. Fourier Transform Infrared (FTIR) Spectra Analysis

**Figure 5:**
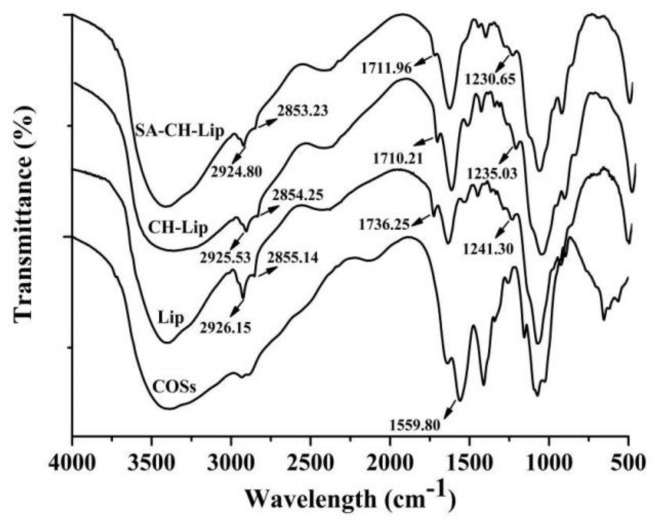
Fourier transform infrared (FTIR) spectra of COSs and COSs nano-liposomes.

The chemical structures of COSs, COSs-Lip, CH-COSs-Lip, and SA/CH-COSs-Lip can be determined by Fourier infrared spectrometer (FTIR), and then the changes of chemical bonds caused by modification can be deduced, which could prove that the outer layer of COSs-Lip was successfully coated with chitosan and sodium alginate, as Figure 5 shows. The liposomal phospholipid bilayer interface can be characterized by these two polar groups, C=O and P=O. In the spectrum of COSs-Lip, 1736.25 cm^−1^ was the stretching vibration of C=O [34], and 1241.30 cm^−1^ was the symmetric vibration of P=O [35], while the absorption peaks at 2926.15 and 2855.14 cm^−1^ are symmetric and asymmetric CH_2_ stretching vibrations of propylene chains in the bilayer of liposomes [36]. To compared the spectra of COSs-Lip, CH-COSs-Lip and SA/CH-COSs-Lip, the symmetric and asymmetric CH_2_ stretching vibrations of propylene remain basically unchanged in CH-COSs-Lip (2925.53 cm^−1^ and 2854.25 cm^−1^) and SA/CH-COSs-Lip (2924.80 cm^−1^ and 2853.23 cm^−1^). However, the absorption peaks representing C=O moved to 1710.21 cm^−1^ in CH-COSs-Lip and 1711.96 cm^−1^ in SA/CH-COSs-Lip respectively. It suggested that the new hydrogen bonds are formed or strengthened during the modification of liposomes [37]. In addition, the absorption peaks represented by P=O group appeared at 1235.03 cm^−1^ in CH-COSs-Lip and 1230.65 cm^−1^ in SA/CH-COSs-Lip, which indicated the formation of new hydrogen bonds between chitosan and nano-liposomes, and proved that liposomes were coated with chitosan successfully. The characteristic peak of amino group in chitosan appeared at 1655 cm^−1^, and the characteristic peak of asymmetric stretching vibration of carboxyl group in sodium alginate appeared at 1614 cm^−1^. These characteristic peaks disappeared in CH-COSs-Lip and SA/CH-COSs-Lip, indicating that chitosan and sodium alginate interacted with each other. The sodium alginate successfully was coated on CH-COSs-Lip.

#### 3.1.5. XRD Analysis

**Figure 6:**
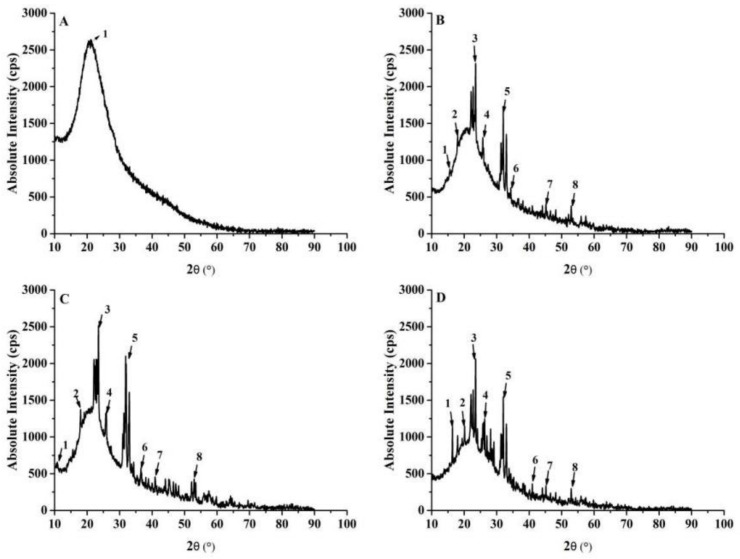
XRD diffraction patterns of COSs and COSs nano-liposomes. (**A**) COSs. (**B**) COSs-Lip. (**C**) CH-COSs-Lip. (**D**) SA/CH-COSs-Lip.

Self-assembly of liposomes is a physical transformation. It is generally believed that the phospholipid bilayer of liposomes fits to the embedded flow model, which means that the bilayer of coated liposomes has crystal properties and the overall crystallinity of the system can be enhanced. The diffraction intensity increased after the formation of liposomes. X-ray diffraction analysis is used to study the relationship among X-ray, crystal, and compound structure. Figure 6A is the XRD pattern of COSs. Within the range of 0–50 °, only “1” corresponds to A characteristic “hump” at 20.48°, which indicated that COSs are in an amorphous form. The diffraction peak in XRD pattern of COSs-Lip powder was very clear. The diffraction angle (2θ) of crystalline peak shown in Figure 6B was at 16.98°, 19.74°, 25.74°, 25.98°, 31.32°, 32.02°, 43.58°, and 52.16°, while the diffraction peak disappeared at 20.48°. This indicated that the COSs were encapsulated by nano-liposomes formed by lecithin and cholesterol. However, there were also larger lumps corresponding to amorphous regions, and it declared liposome powder was semi-crystalline. The diffraction characteristic peaks of lecithin and cholesterol are mostly from 30° to 50°. Figure 6C shows the CH-COSs-Lip was wrapped by chitosan. In the XRD curve of CH-COSs-Lip, the characteristic diffraction peak of 16.98° of COSs-Lip has shifted by 6.84°, and the positions of other characteristic peaks have no significantly different from that of COSs-Lip.

Compared with COSs, COSs-Lip, and CH-COSs-Lip, the baseline XRD of SA/CH-COSs-Lip was stable, with narrow diffraction peak and high intensity, indicating relatively high crystallinity and good regularity. According to Figure 6D, the crystal diffraction peaks in SA/CH-COSs-Lip correspond to the diffraction peaks at 18.78°, 22.12°, 24.08°, 25.08°, 32.04°, 40.14°, 45.40°, and 52.28° for 2θ, respectively, while the diffraction peaks disappear at 6.84° and 20.48°. Compared with the diffraction peaks of COSs and CH-COSs-Lip, the shift may be led by sodium alginate as an embedding material that confined the COSs in a very small space and inhibited the movement of the carbon–hydrogen bond in COSs [38]. This result was consistent with the results of infrared spectrum. In addition, the results also indicated that, in the vacuum freeze-drying process, sodium alginate enters into CH-COSs-Lip and combines with the lipid film to produce a new crystal phase, which can effectively protect liposomes.

### 3.2. In Vitro Simulated Digestion Analysis

During the gastrointestinal digestion, nano-liposomes are easily destroyed by strong acidic and alkaline digestive juices, resulting in the leakage of embedded substances in liposomes and reducing their bioavailability [39]. In order to characterize the effect of the coated liposomes on the transport and absorption of COSs, the liposomes carrying COSs before and after the modification were subjected to in vitro simulated digestion experiments.

**Figure 7:**
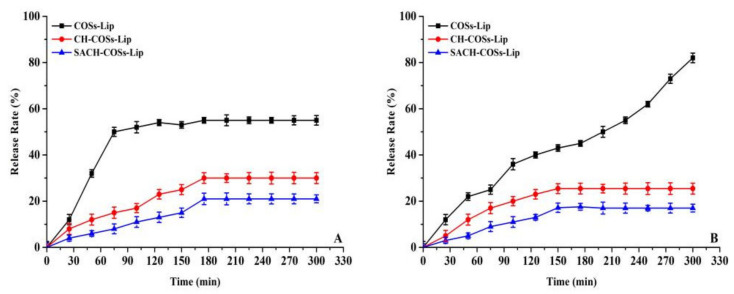
Release rate of COSs from COSs nano-liposomes during simulated intestinal digestion. (**A**) Simulated gastric digestion. (**B**) Simulated intestinal digestion.

According to Figure 7A, the release of COSs in COSs-Lip, CH-COSs-Lip, and SA/CH-COSs-Lip simulated gastric digestion in vitro showed a trend of “fast followed by slow” type, but the release rate of uncoated COSs-Lip was significantly higher than that of the two modified liposomes. After 75 min of gastric digestion, the release rate of COSs-Lip, CH-COSs-Lip, and SA/CH-COSs-Lip was 50.14%, 14.92%, and 8.22%, respectively. When the digestion time was extended to 175 min, these three kinds of liposomes basically reached the highest COSs release rate, and the release rates of COSs-Lip, CH-COSs-Lip, and SA/CH-COSs-Lip were 55.02%, 31.21%, and 21.13%, respectively. After that, there was no significant change in the release rate of COSs among these three liposomes. During the process of gastric digestion, the release rate of COSs in COSs-Lip was much higher than that of CH-COSs-Lip and SA/CH-COSs-Lip. This may be because sodium alginate can reduce the activity of pepsin, and it has been shown that chitosan in stomach acid can reduce the release of drugs [40]. Therefore, SA/CH-COSs-Lip has higher stability in gastric juice, which is conducive to the transport of more embedded COSs to the small intestine.

As can be seen from Figure 7B, in the process of simulated intestinal digestion in vitro, the release rate of COSs encapsulated in COSs-Lip showed a trend of constant increase over time; when the digestion time was 300 min, the release rate reached to 82.81% ± 1.73%. In contrast, CH-COSs-Lip and SA/CH-COSs-Lip showed a trend of rapid release followed by slow release; when digestion time reached to 150 min, CH-COSs-Lip and SA/CH-COSs-Lip basically reached the maximum COSs release rates, which were 25.44 ± 1.14% and 17.18 ± 0.63%, respectively. Thereafter, there was no significant change in the release rate of COSs between these two liposomes. Thus, unmodified liposomes release more COSs than coated liposomes during the entire process of simulating intestinal digestion.

The release rate of COSs in intestinal fluid of liposomes was significantly higher than that in gastric fluid. This may be due to the hydrolytic effect of trypsin in intestinal fluid on the outer layer of phospholipids that leads to the hydrolytic rupture of liposomes and the release of a large amount of COSs. However, chitosan and sodium alginate on the surface of liposomes can prevent trypsin in intestinal fluid from contacting with phospholipids in the outer layer of liposomes, thus protecting the integrity of liposomes and making the coated COSs difficult to exudate. Nano-liposomes can be absorbed through the mucosal layer on the surface of the intestine, and it has been found that nanoparticles can be absorbed directly by epithelial cells through active or passive transport mechanisms. Therefore, the layer-by-layer coated liposomes can greatly improve the bioavailability of COSs. Moreover, with the COSs released during the simulated intestinal digestion process, we deduce that the chemical structure of COSs could change, e.g., the β-1,4-glycosidic linkages break and degree of polymerization is reduced.

### 3.3. Cell Experiment

#### 3.3.1. MTT Assay Results

**Figure 8:**
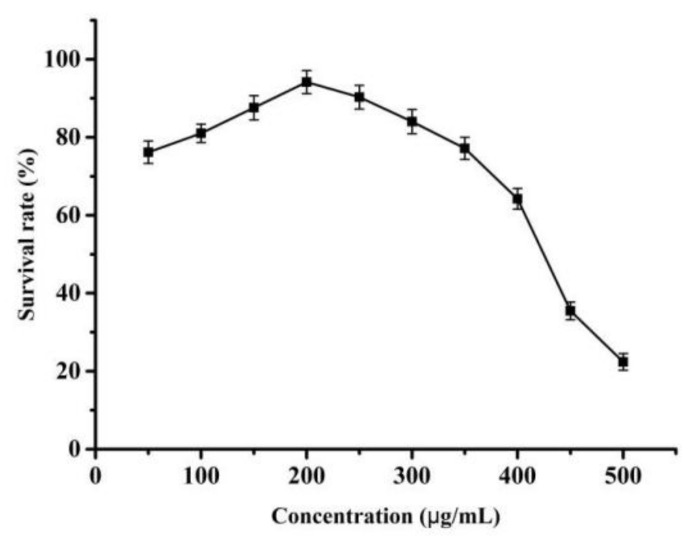
Effect of COSs nano-liposomes on the survival rate of Caco-2 cells model.

The effects of the nano-liposomes of different concentrations on the survival rate of Caco-2 cells were investigated by the MTT assay, and the survival rate of Caco-2 cells is shown in Figure 8. As shown, the Caco-2 cell survival rate increased to 96.15% at a nano-liposomes concentration of 200 µg/mL and then decreased gradually when the concentration of the nano-liposomes increased. Based on this finding, when the concentration was lower than 200 µg/mL, the survival rate of Caco-2 cells was greater than 90% after incubation with nano-liposomes for 24 h, with no evident cytotoxicity. Considering the encapsulation efficiency of the nano-liposomes, the selected nanogel concentration was 200 µg/mL for the subsequent Caco-2 cell transport experiment.

#### 3.3.2. Transport Rate of the Nano-Liposomes onto Caco-2 Cells

**Figure 9:**
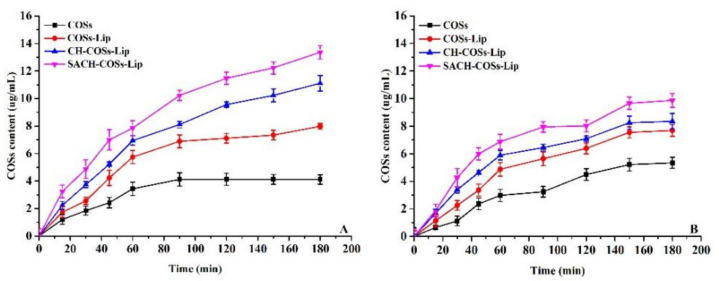
Time–effect relationship of COSs nano-liposomes transport on Caco-2 cells model. (**A**) From AP side to BL side. (**B**) From BL side to AP side.

Figure 9 displays different trends of the transport rate of the nano-liposomes in the monolayer model of Caco-2 cells over time, from the AP side to the BL side and from the BL side to the AP side. With the time extension, the transfer volume of nano-liposomes in both directions was increased. Thereby, it demonstrated the obvious dependence of these bidirectional transfers on time. The penetration of nano-liposomes showed a linear increase within 120 min and reached the saturation point at 150 min. It may be caused by the lack of a concentration gradient and some active carriers reaching the saturation point. It suggests that active transport and passive transport exist simultaneously. From the AP side to the BL side, the transport content of nano-liposomes was greater than that in the reverse direction. The transport content of nano-liposomes from the AP side to the BL side within 3 h was also significantly higher than the other way around. This indicated that the transport of the core–shell nanogels was directional and was excreted by a transport protein. From the cumulative transport content, nano-liposomes in the Caco-2 cell monolayer were greater than those in exocytosis. In both directions, the apparent permeability coefficient was greater than 1.0 × 10^−6^ cm/s (Table 2). Furthermore, a significant difference was found between the groups (*p* < 0.05), which means the nano-liposomes were better absorbed in intestine.

The transshipment quantity of nano-liposomes from the AP side to the BL side was significantly larger than that of the enzymatic hydrolysate on CaCo-2 cell monolayer. The apparent permeability coefficient (at 120 min) was significantly higher than that of the latter. This phenomenon was mainly caused by SA/CH, which served as the nano-embedded materials and formed a protection for the nano-liposomes. As a result, the degradation of nano-liposomes by proteases in the digestive tract is reduced and the absorption effect in the human digestive system is effectively improved. After SA/CH embedding, the absorption efficiency may be improved through its vector-targeting mediated pathway. There was no significant difference in apparent permeability coefficient between nano-liposomes from the BL side to the AP side (Table 2). However, the reduction of the efflux effect should be examined in a further study.

## 4. Conclusions

In summary, chito-oligosaccharides (COSs) nano-liposomes were prepared by the film-ultrasonic method, and the nano-liposomes were successfully layer-by-layer coated with chitosan and sodium alginate, which were uncoated liposomes (COSs-Lip), chitosan-coated liposomes (CH-COSs-Lip), and sodium alginate (SA)/chitosan (CH)-coated liposomes (SA/CH-COSs-Lip). The CH-COSs-Lip and SA/CH-COSs-Lip exhibited a core–shell structure in TEM and signal changes of characteristic peaks in FTIR. Moreover, SA/CH-COSs-Lip was obtained as a smaller-sized distribution with a higher encapsulation efficiency than uncoated CH-COSs-Lip and COSs-Lip. Furthermore, a quantitative estimate of in vitro simulated digestion stability suggested that chitosan and sodium alginate layer-by-layer coated could better prevent COSs released than COSs-Lip and CH-COSs-Lip. In comparison to COSs-Lip and CH-COSs-Lip, SA/CH-COSs-Lip exhibited a higher cellular uptake and transport in Caco-2 cells. Thus, the results of this study provide scientific evidence for the application of COSs nano-liposomes modified by sodium alginate and chitosan in the food field.

## Figures and Tables

**Table 1:** Encapsulation efficiency, D_Z_, and PDI of COSs and different nano-liposomes.

Group	Dz (nm)	PDI	Encapsulation Efficiency (%)
COSs	187.30 ± 5.20 ^a^	0.118 ± 0.097 ^a^	–
COSs-Lip	201.44 ± 4.68 ^b^	0.227 ± 0.145 ^b^	61.23 ± 2.09 ^c^
CH-COSs-Lip	256.47 ± 5.62 ^c^	0.283 ± 0.134 ^c^	82.34 ± 4.53 ^b^
SA/CH-COSs-Lip	331.19 ± 6.75 ^d^	0.331 ± 0.085 ^d^	94.45 ± 3.08 ^a^

Data were expressed as the mean value (*n* = 3) ± standard deviation. ^a, b, c, d^ in the same column indicated a significant difference (*p* < 0.05).

**Table 2:** Apparent permeability coefficient of two-way transport of COSs and different nano-liposomes.

Group	PappAB (×10^−6^ cm/s)	PappBA (×10^−6^ cm/s)
COSs	14.46 ± 0.34 ^d^	4.31 ± 0.29 ^a^
COSs-Lip	20.07 ± 2.65 ^c^	4.87 ± 0.34 ^a^
CH-COSs-Lip	24.44 ± 1.57 ^b^	4.90 ± 0.43 ^a^
SA/CH-COSs-Lip	28.89 ± 3.41 ^a^	4.76 ± 0.28 ^a^

Data were expressed as the mean value (*n* = 3) ± standard deviation. ^a, b, c^^, d^ in the same column indicated a significant difference (*p* < 0.05).

## Data Availability

Research data are not shared.
